# New Strategy for a Suitable Fast Stabilization of the Biomethanization Performance

**DOI:** 10.1155/2012/418727

**Published:** 2012-11-05

**Authors:** L. A. Fernández-Güelfo, C. J. Álvarez-Gallego, D. Sales Márquez, L. I. Romero García

**Affiliations:** ^1^Department of Chemical Engineering and Food Technology, Faculty of Science, University of Cadiz, Cadiz, 11510 Puerto Real, Spain; ^2^Department of Environmental Technologies, Faculty of Marine and Environmental Sciences, University of Cadiz, Cadiz, 11510 Puerto Real, Spain

## Abstract

The start-up strategies for thermophilic anaerobic reactors usually consist of an initial mesophilic stage (35°C), with an approximate duration of 185 days, and a subsequent thermophilic stage (55°C), which normally requires around 60 days to achieve the system stabilizatio. During the first 8–10 days of the mesophilic stage, the reactor is not fed so that the inoculum, which is generally a mesophilic anaerobic sludge, may be adapted to the organic solid waste. Between mesophilic and thermophilic conditions the reactor is still not fed in an effort to prevent possible imbalances in the proces. As a consequence, the start-up and stabilization of the biomethanization performance described in the literature require, at least, around 245 days. In this sense, a new strategy for the start-up and stabilization phases is presented in this study. This approach allows an important reduction in the overall time necessary for these stages in an anaerobic continuous stirred tank reactor (CSTR) operated at thermophilic-dry conditions for treating the organic fraction of the municipal solid waste (OFMSW): 60 days versus 245 days of conventional strategies. The new strategy uses modified SEBAC technology to adapt an inoculum to the OFMSW and the operational conditions prior to seeding the CSTR.

## 1. Introduction

The organic fraction of municipal solid wastes (OFMSW) has been commonly treated by means of anaerobic digestion (AD) [1–3]. Among the main advantages of this biological process, the low energy consumption and sludge generation and the high hydrogen and/or methane productions must be highlighted; however, the main disadvantage is its slowness.

In order to avoid this important inconvenience and to accelerate the process with regard to the mesophilic-wet conditions (35°C, 5–10% total solids concentration), AD may be operated at thermophilic-dry (55°C, 30% total solids concentration) conditions. At these new conditions the hydrolysis phase is faster and more effective and, therefore, the overall rate of the process is improved. On the other hand, it must be noted that for the start-up and stabilization of the biomethanization process in continuous stirred tank reactor (CSTR) for anaerobic biodegrading of OFMSW is necessary operational times extremely long. 

As it is reported by the authors Bolzonella et al. [4] and Michaud et al. [5], the strategy generally employed to start-up and stabilization of thermophilic anaerobic digesters consists of two stages.

1. A mesophilic stage (35°C) of about 185 days of operation. During the first 8–10 days the reactor is not fed in order to the inoculum (generally anaerobic mesophilic sewage sludge) may be adapted to the waste.
2. A thermophilic stage (55°C) with an approximate duration of 60 days at least. In addition, during the mesophilic-thermophilic transition the reactor is not fed to prevent destabilization episodes.

Following this, the strategies reported in the literature have an approximate duration of about 245 days at least. This fact is mainly because the inoculum is not adapted to the type of waste and/or the operational conditions.

With specific reference to the full-scale industrial application of the AD processes, at first, because of the historical background, more reactors adopting the wet processes (<10% dry solids in the reactor) were applied; since then, dry digestion (more than 25% dry solids in the feed) has prevailed because of the reduced volume of reactors and wastewater production. Most applied technologies for dry processes are Dranco, Valorga, Linde, and Kompogas, all working in the range 30–40% of total solids in the reactor feeding [6]. 

About the conventional ranges of temperature of the AD processes, a total treatment capacity for solid waste organics, excluding the tonnage used for sewage sludge and manures, evolved from 122,000 ton per year in 1990 to 1,037,000 ton available or under construction by the last decade in 53 plants across Europe, an increase by 750%. Both mesophilic and thermophilic technologies have been proven, with about 38% of capacity being operated at thermophilic temperatures. All digestion plants were initially operated at mesophilic temperatures. The first thermophilic plants were dry fermentation plants and came online in 1992 and 1993. The capacity of mesophilic operation increased by 350,000 ton during 1994 through 1999, while thermophilic capacity increased by 280,000 ton or 70,000 ton and 56,000 ton per year, respectively. During some years, more mesophilic plants are added while during other years more thermophilic capacity is constructed. No clear trend can be observed. It can be expected that the increase will be level for both temperature ranges, even though more suppliers are starting to provide thermophilic digestion. Thermophilic operation was developed later but has been established as a reliable and accepted mode of fermentation. It provides the added benefit of treating the waste at higher temperatures and thereby increasing pathogen kill-off during the anaerobic phase. The added amount of heat does not seem to stop companies operating thermophilically, as higher gas production yields and rates are being claimed by various suppliers [7].

For all the above reasons, an inoculum adapted to the waste (OFMSW) and the operational conditions (thermophilic-dry AD) was obtained by mean of the modified sequencing batch anaerobic composting (SEBAC) technology. This technology and its modifications are fully detailed in the literature [8–11] and have been successfully employed to develop the AD of OFMSW with acceptable conversions in only 30 days.

The modification of the SEBAC technology used in this research is based on the interconnection of two anaerobic digesters (reactors A and B). Daily, reactors A and B are fed by means of the recirculation of the leachate generated from the other reactor. In this way the fresh organic waste (reactor A) to be digested is inoculated through recirculation of the leachate or effluent from the reactor containing the digested waste (reactor B), while the leachate generated by the reactor with fresh waste (reactor A) is recirculated to the reactor with the digested waste (reactor B). In this way a flow of microorganisms is established to the undigested waste and of organic material to the digested waste [12].

Based on all the stated above and in order to reduce the long-time periods required for the start-up and stabilization of the biomethanization in a CSTR operated at thermophilic-dry conditions for treating OFMSW, two main goals may be defined in this work which are as follows.

- To obtain a suitable inoculum quickly by means of the modified SEBAC technology commented previously. This inoculum will be adapted to the OFMSW and the operational conditions typical of the thermophilic-dry AD (55°C and 30% total solids concentration).
- To achieve stable biomethanization performance for the thermophilic-dry AD of OFMSW in a CSTR using the inoculum obtained by the modified SEBAC technology.

## 2. Materials and Methods

### 2.1. Modified SEBAC Technology

**Figure 1:**
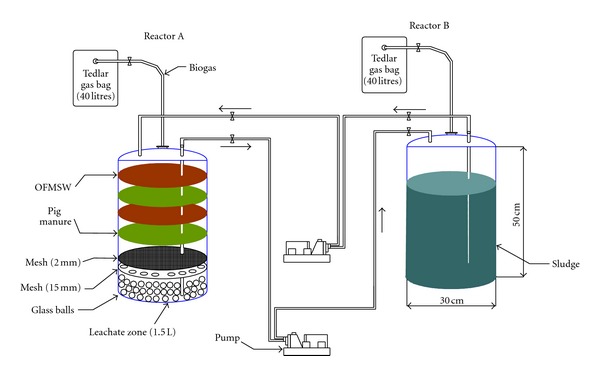
Flow of leachate in the modified SEBAC reactors.

This system consists of the interconnection of two anaerobic 25 L-reactors (Figure 1), operating under thermophilic-dry conditions (55°C and 30% total solids concentration).

- Reactor A contains alternate layers of source selected OFMSW and pig manure with a total solids concentration of 30%. The pig manure accelerates the colonization of the OFMSW since it is a potential source of anaerobic microorganisms.
- Reactor B generally contains a stabilized waste previously degraded by anaerobic digestion, with a high concentration of viable and active microorganisms [13]. In this study, anaerobic mesophilic sludge, from the anaerobic digesters of a full-scale plant for treating sewage sludge, was used.

Daily, the leachate produced in reactor A was exchanged to reactor B and an equivalent quantity of sludge from reactor B was added to reactor A. This procedure causes a flow of microorganisms from B to A and a flow of organic matter from reactor A to B. Reactors do not require agitation and the time required for effective start-up is around 30 days. The composition of the modified SEBAC reactors is shown in Table 1.

### 2.2. Continuous Stirred Tank Reactor (CSTR)

The CSTR was initially loaded with 1.5 kg of milled and dry synthetic OFMSW (90% in total solids concentration). The moisture was adjusted using the inoculum obtained by means of the modified SEBAC technology described above. Concretely, the inoculums consisted of a 1 : 1 v/v mixture [14–16] of thermophilic sludge and leachate. In this sense, 4 litres of inoculum (2 litres of sludge + 2 litres of leachate) were required to add moisture to the synthetic OFMSW.

The compositions of the different wastes used in this study are given in Table 2. It must be noted that the synthetic OFMSW was prepared based on the nutritional requirements of the main populations of microorganisms involved in the AD [17]. This type of feed avoids the problem of high variations in the composition of the source selected OFMSW. This aspect is important in order to determine an accurate efficiency for the process.

### 2.3. Processing of the OFMSW

Control of the total solid concentration of the feed is necessary to obtain a suitable level of performance for the dry AD. Therefore, pretreatment of the OFMSW samples was necessary to adjust them to the required optimum values. In this study, the samples were dried at 55°C for 48 hours and then at ambient temperature for 72 hours until final moisture content of 10% was achieved. The dried OFMSW was milled until a particle size of approximately 1 cm was obtained and, finally, the moisture was adjusted to 70–75% (25–30% in total solids concentration, which is characteristic of dry AD) with tap water, leachate from garbage, sludge, or combinations of these.

### 2.4. Analytical Techniques

For the control of the reactors, the following parameters were determined: the volume and composition of the biogas (H_2_, O_2_, N_2_, CH_4_, and CO_2_), volatile fatty acids (VFA), total solids (TS), suspended total solids (STS), total volatile solids (TVS), suspended volatile solid (SVS), alkalinity, pH, dissolved organic carbon (DOC), ammonium, chemical oxygen demand (COD), and density. The analytical techniques were performed according to procedures described by Álvarez-Gallego [18].

## 3. Results and Discussion

### 3.1. Inoculum Preparation through Modified SEBAC Technology

The daily volume of leachate exchanged between the two modified SEBAC reactors must be between 5 and 10% of the initial volume of OFMSW for digestion [8]. For this reason, Reactor A controls the start-up phase, which is the *rate limiting step* of the process [19]. The theoretical calculation indicates that the volume of leachate that should be exchanged is approximately 600 mL. From the fourth day of operation until the conclusion of the experiment (hundredth day), the flow of leachate between the two reactors was maintained at 600 mL.

**Figure 2:**
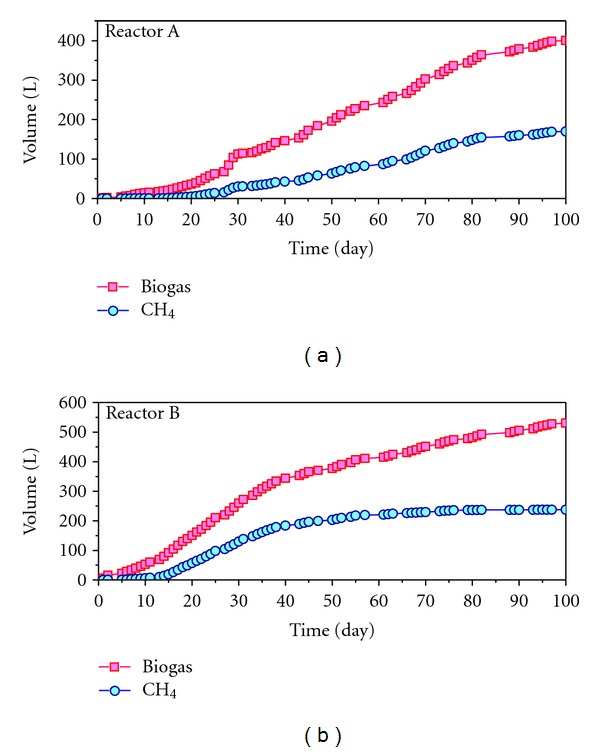
Accumulation of biogas and methane productions in the modified SEBAC reactors.

The minimum time required to obtain a suitable inoculum through modified SEBAC technology may be determined from the accumulated methane production curves generated in reactors A and B. As can be seen in Figure 2, the curves of both reactors present the maximum slope in 30 days of operation, more pronounced in reactor B (sludge). This fact indicates an exponential growth of the methanogenic Archaea in the system and, therefore, if the inoculum is taken in this moment, it will present a high methanogenic activity. Thus, the sludge from day 30 can be considered as a viable inoculum for the biomethanization of OFMSW at thermophilic-dry conditions. Finally, the initial and final compositions of wastes at the end of the assay are shown in Table 3.

### 3.2. Start-Up and Stabilization of the Biomethanization Process

A continuously stirred tank reactor (CSTR) was started-up under thermophilic-dry conditions and a series of four solid retention times (SRT) was carried out in order to study the effect of the added organic loading rate (OLR_0_) on the biomethanization performance at semicontinuous regime of feeding.

Along the four consecutive stages, the OLR_0_ (expressed as mgDOC/L·d and mgTVS/L·d) was increased and it was maintained constant in each SRT. The SRT, OLR_0_, and operation time data for each stage are shown in Table 4.

In the first stage the OLR imposed was relatively low (0.704 gDOC/L·day) in order to check if the system evolved appropriately. The results obtained in the first 14 days were favourable and, therefore, the OLR was increased to 0.805 gDOC/L·day. The OLR_0_ used at stage 1 was different to the values reported in the literature. Bolzonella et al. [4] carried out start-up phase studies in the mesophilic range with an extremely low OLR—less than 0.16 gDOC/L·day—for approximately 40 days. It must be highlighted that, in this study, the start-up phase was carried out using the SEBAC inoculum, which had been previously adapted to the waste and operational conditions. This fact allows that the reactor may be operated at higher OLR.

#### 3.2.1. Study of the Gas Productions

**Figure 3:**
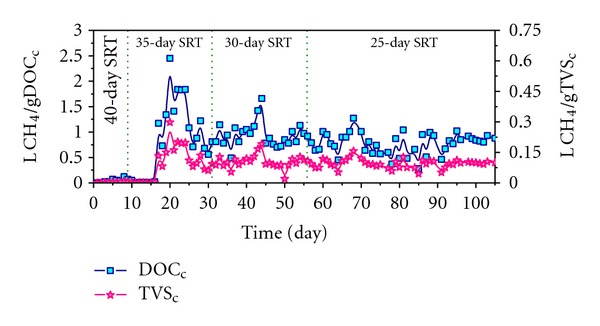
Evolution of the specific methane yield expressed as LCH_4_/gDOC_c_ and LCH_4_/gTVS_c_.

As can be seen from Figure 3, the biogas generated in stage 1 is not useful and this stage may be considered as a latency period in which the hydrolysis and colonization of the waste takes place. During stage 2, the specific methane yield reaches its maximum average values of 1.11 LCH_4_/gDOC degraded and 0.51 LCH_4_/gTVS degraded due to the biodegradation of the VFA accumulated in the previous stage. Finally, during stages 3 and 4, the methane yield coefficient stays constant at around 0.91 LCH_4_/gDOC and 0.1 LCH_4_/gTVS respectively, indicating stable biomethanization performance in the system.

On the other hand, the average specific methane yield in term of COD reaches the value of 0.42 LCH_4_/gCOD in stage 2 and 0.34 LCH_4_/gCOD in stages 3 and 4. However, in stage 1 the average specific methane yield is practically zero, 0.01 LCH_4_/gCOD. In accordance with Bushwell and Mueller [20], the stoichiometric value for methane generation is 0.35 LCH_4_/gCOD, which indicates that in stages 3 and 4 the reactor is working with a methane yield coefficient very close to the theoretical maximum. However, the value obtained for the 35-day SRT (stage 2) is higher than the theoretical maximum. This discrepancy is due to the fact that in this SRT, in addition to the degradation of the OLR added, the transformation of the organic matter accumulated in the system during the previous 40-day SRT takes place. As a consequence, the methane yield coefficient obtained is higher than its theoretical value.

**Figure 4:**
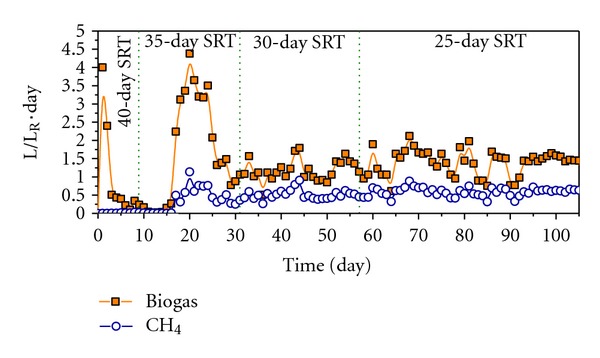
Daily biogas and methane productions expressed as L/L_Reactor_ *·*day.

**Figure 5:**
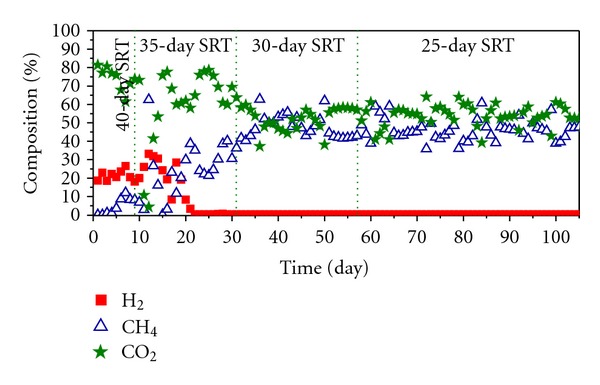
Biogas composition expressed as percentage.

As far as the daily biogas generation is concerned, during the first 3 days of stage 1 a significant level of production was observed due to the hydrolysis of the waste (Figure 4). The composition observed in this period is usual for hydrolytic phase: H_2_ (20%) and CO_2_ (80%), see Figure 5. 

During the hydrolysis phase complex molecules are transformed into other simpler products, without methane production. For this reason, in Figure 5, stage 1 has been considered as a latency phase.

However, in stage 2, H_2_ levels drop to zero due to the methanogenic activity while CH_4_ and CO_2_ converge at around 50%, which is typical behaviour of a stable biomethanization process. In this phase, the intermediate products generated in the hydrolysis are converted into CO_2_ and CH_4_ by the methanogenic Archaea and as consequence the daily average biogas production reaches its maximum average value of 1.834 L_Biogas_/L_Reactor_·day an average stable methane production of 0.55 LCH_4_/L_Reactor_·day after 15 days of operation time. About the last value, 0.55 LCH_4_/L_Reactor_·day, it is higher than the result reported by Fernández et al. [21] in their dry AD studies of OFMSW using the same technology (CSTR) with similar SRTs. In that work, sewage sludge anaerobically digested at mesophilic regime of temperature coming from a full-scale WWTP was used as inoculums to start-up a CSTR. In that case, the system reaches a stable methane production of 0.48 LCH_4_/L_R_ *·*day after 25 days of operation time and at 30-day SRT. In this work, the system reaches a stable methane production of 0.55 LCH_4_/L_R_ *·*day after 15 days of operation time and at 35-day SRT (very positive result since higher SRTs are associated with low methane yields). Therefore, the start-up period is decreased about 40% with this strategy and, in addition, the methane production is improved around 15% although the SRT is higher (35 versus 30 days), and, therefore, the methane production should be lower.

In stage 3 the daily average biogas production decreases to 1.138 L_Biogas_/L_Reactor_·day since most of the initial waste which was loaded into the reactor has been degraded. Nevertheless, the composition of the biogas is stabilized with values of CO_2_ and CH_4_ at around 50%, which indicates that a balance between the different microbial populations involved in the digestion has been reached in the reactor.

Finally, in stage 4 the daily average biogas production increase to 1.768 L_Biogas_/L_Reactor_·d due to the OLR_0_ increased, with a composition in CO_2_ and CH_4_ of 55 and 45%, respectively (Figure 5). 

#### 3.2.2. VFA Evolution

**Figure 6:**
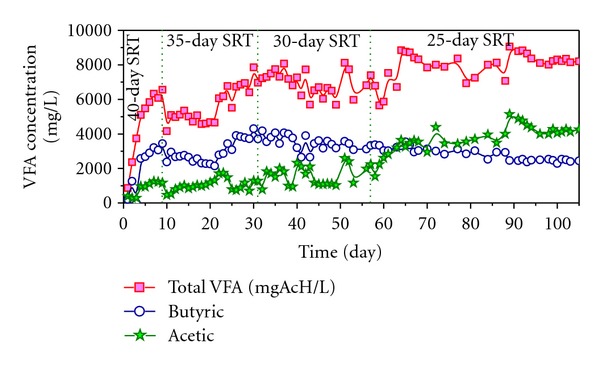
Evolution of total VFA, butyric, and acetic acids.

As can be seen in Figure 6, from day 90 at 35-day SRT, the total VFA and butyric and acetic acids concentrations reach very stable values of around 8000, 2500, and 400 mg/L, respectively. It must be noted that in this specific period (from 90 to 105 days), the stabilization of VFA concentrations matches with a stable specific methane yield, expressed in terms of DOC and TVS (Figure 3), and with a stable biogas and methane productions (Figure 4). In addition, removal percentages of 56% for TS, 89% for TVS, and 63% for DOC were observed. These values have been compared with literature values [8, 22] and they confirm that the biomethanization operates efficiently at stable conditions. Hence, it is possible to reach stable biomethanization performance in SRT that is appropriate for full-scale plants (25 days) in approximately 90 days.

## 4. Conclusions

As a general conclusion, a successful strategy for the start-up and stabilization phase of the biomethanization process of OFMSW in a CSTR operated at thermophilic-dry conditions has been developed. The new strategy allows stable operation in a reduced time versus other literature protocols. Taking into account the above main conclusion, the following specific conclusions may be established.

1. In the first stage, a thermophilic anaerobic inoculum adapted to the OFMSW must be obtained by means of the modified SEBAC technology. This inoculum is used in the second stage to inoculate the CSTR. The semicontinuous reactor must be subsequently fed with milled OFMSW in a high SRT (40 days). When the system is stabilized, the SRT imposed can be progressively diminished until reaching 25-day SRT.
2. The results obtained from the modified SEBAC reactors indicate that an incubation period of approximately 30 days is necessary to obtain an appropriate inoculum. From the day 30 of operation the system reaches a high biogas production with a high methane percentage.
3. The semicontinuous reactor can be inoculated with a 1 : 1 mixture of the effluents (leachate of OFMSW and sludge) from the modified SEBAC reactors and a period for the acclimatization of microorganisms is not required prior to feeding the system.
4. A high retention time must be initially imposed (40 days) to avoid irreversible distortions in the process. The SRT is subsequently reduced progressively, which is associated with an increase in the OLR_0_, until the required operational conditions are reached. The stabilization of the system, for an SRT of 35 days (OLR_0_ of 0.805 gDOC/L·d), requires 30 days of operation. Under these conditions the maximum average methane productions is reached, 1.834 L_Biogas_/L_Reactor_·day.
5. For a successful start-up of the system, it is necessary a period of 60 days at least (30 days to obtain the inoculum and 30 days to stabilize the system at 35-day SRT. In addition, it is possible to reach stable operation in times that are appropriate for industrial operation (25-day SRT) in approximately 90 days. These data compare favourably with literature results for similar reactors, where the start-up periods are higher than 245 days.

In summary, the preparation of an inoculum adapted to the solid waste and operational conditions by means of modified SEBAC procedure enables us to reduce the time necessary to start-up and stabilization of a CSTR for the thermophilic-dry AD of OFMSW by a factor of four (60 days versus 245) with respect to conventional strategies reported in the literature.

## Figures and Tables

**Table 1:** Composition of the modified SEBAC reactors.

	Wastes	Layers	Weight/layer (kg)
Reactor A	OFMSW	2	1
	Pig manure	2	1.5

Reactor B	Sludge	—	21

**Table 2:** Composition of the wastes used to start-up the CSTR.

Parameter	Leachate inoculum	Sewage sludge inoculum	OFMSW	OFMSW/Inoculum mixture
pH	8.62	8.35	7.78	8.70
Density (kg/m^3^)	980	985	750	1116
Alkalinity (gCaCO_3_/L)	21.78	16.54	4.29	5.14
Ammonium (gNH_3_–N/L)	26.88	14.56	1.68	2.8
Total Nitrogen	25.66 gNH_3_–N/L	21.46 gNH_3_–N/L	207.2 gNH_3_–N/kg	72.8 gNH_3_–N/kg
gTSS/L	14.46	20.46	—	—
gVSS/L	10.73	9.16	—	—
gTS/g sample	—	—	0.90	0.31
gTVS/g sample	—	—	0.71	0.25
Total carbon (mg/g)	80.78	35.27	112.6	65.07
Total inorganic carbon (mg/g)	2.07	0.96	0.29	0.30
Total organic carbon (mg/g)	78.41	34.31	112.3	64.75
Acidity (mgAcH/L)	12403	17353	1440	356

**Table 3:** Initial and final compositions of the wastes.

Parameters	OFMSW		Pig manure		Sludge	
	Initial	Final	Initial	Final	Initial	Final
Density (kg/m^3^)	600	850	1200	1000	900	1000
Total solids (g/kg)	878	173	586	80	42	26.6
Total volatile solids (g/kg)	700.4	85	464.1	60	15	2.6
Suspended total solids (g/L)	0.5	7.9	3.9	5	20.2	11.4
Suspended volatile solids (g/L)	3.6	6.9	3.5	3.4	7.7	7.4
pH	0.2	8.1	7.1	8.4	8.3	8.35
Alkalinity (gCaCO_3_/L)	7.6	8.4	50	75.4	20.1	16.5
Chemical oxygen demand (mgO_2_/L)	112000	41558	14814	6509	10527	25526

**Table 4:** Initial organic loading rate (OLR_0_) for each SRT.

Stage	SRT (day)	Operation time (day)	OLR_0_	
			gDOC/L·day	gTVS/L·day
1	40	14	0.704	4.42
2	35	17	0.805	5.07
3	30	25	0.940	5.92
4	25	50	1.123	7.50
